# Streptoquinolines A and B, new antibacterial meroterpenoids produced by *Streptomyces* sp. TMPU-A0679

**DOI:** 10.3762/bjoc.22.12

**Published:** 2026-01-27

**Authors:** Akiho Yagi, Hitomi Tomura, Ami Konno, Ryuji Uchida

**Affiliations:** 1 Division of Natural Product Chemistry, Faculty of Pharmaceutical Sciences, Tohoku Medical and Pharmaceutical University, 4-4-1 Komatsushima, Aoba-ku, Sendai, Miyagi 981-8558, Japan

**Keywords:** meroterpenoid, quinoline, *Streptomyces* sp, streptoquinoline, VRE

## Abstract

In the course of screening vancomycin-resistant enterococci (VRE), two new meroterpenoids, streptoquinolines A (**1**) and B (**2**), were isolated from a culture of terrestrial *Streptomyces* sp. TMPU-A0679. The structures of **1** and **2** were elucidated based on spectroscopic analyses, including NMR and MS, and were found to consist of a drimane-type sesquiterpene fused with a highly substituted quinoline moiety. Further structural comparisons and specific rotation data revealed that **1** and **2** were epimers at C-4'. Compounds **1** and **2** both exhibited selective antibacterial activity against Gram-positive bacteria, including VRE and methicillin-resistant *Staphylococcus aureus*, with minimum inhibitory concentrations ranging from 6.25 to 12.5 µg/mL. These results expand the structural and biological diversities of drimane–quinoline-type meroterpenoids and highlight their potential as lead compounds for the development of new antibacterial agents targeting drug-resistant Gram-positive pathogens.

## Introduction

The emergence of antimicrobial-resistant bacteria has become a serious public health issue worldwide. Infections caused by these pathogens severely limit available treatment options and contribute to increased mortality rates [1]. Vancomycin-resistant enterococci (VRE), represented by *Enterococcus faecium* and *E. faecalis*, are responsible for infections such as urinary tract infections, bacteremia, and endocarditis, which may lead to severe complications, particularly in elderly or immunocompromised patients [2]. Current treatment relies on only a few agents, including linezolid and daptomycin, and the emergence of resistant strains to these drugs poses an additional clinical challenge [3].

Therefore, the World Health Organization (WHO) revised its Bacterial Priority Pathogens List (BPPL) in 2024, reaffirming VRE as a “High Priority Pathogen” for which research on and the development of new antimicrobial agents are urgently needed [4]. This emphasizes the continuing demand for antibiotics with novel chemical scaffolds and mechanisms of action.

**Figure 1:**
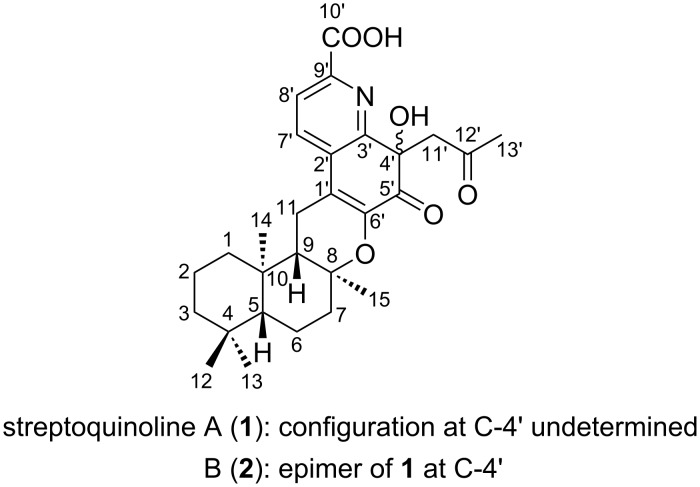
Structures of streptoquinolines A (**1**) and B (**2**).

In our ongoing search for anti-VRE agents from microbial resources, we previously reported antimicrobial compounds such as tirandamycin and micrococcin [5–6]. During this study, we isolated two previously unreported meroterpenoids from a culture of *Streptomyces* sp. TMPU-A0679 and designated them as streptoquinolines A (**1**) and B (**2**) (Figure 1). Compounds **1** and **2** possessed a unique skeleton consisting of a drimane-type sesquiterpene fused with a quinoline moiety and both exhibited potent antibacterial activity against Gram-positive bacteria. This study describes the production, isolation, structural elucidation, and antibacterial activities of compounds **1** and **2**.

## Results and Discussion

The actinomycete strain TMPU-A0679 was isolated from a soil sample collected in Hofu city, Yamaguchi, Japan, and was identified as *Streptomyces* sp. based on a BLAST analysis of its 16S rDNA sequence. This strain was cultivated on a rotary shaker (180 rpm) at 27 °C for 7 days using a malt extract-based production medium. Harvested mycelia (1.0 L) were extracted with acetone and ethyl acetate, and purification was guided by antibacterial activity against VRE. The crude extract (922 mg) was subjected to ODS column chromatography followed by preparative HPLC, yielding streptoquinolines A (**1**, 7.50 mg) and B (**2**, 8.05 mg).

The physicochemical properties of **1** and **2** are summarized in Table 1. Compounds **1** and **2** showed characteristic absorption maxima at 200–202, 214, 231–234, 312, and 349–350 nm in UV spectra. Common IR absorption bands at 3414–3427 and 1620–1680 cm^−1^ indicated the presence of hydroxy and carbonyl groups, respectively.

**Table 1:** Physicochemical properties of **1** and **2**.

	**1**	**2**

appearance	yellow oil	yellow oil
molecular weight	481	481
molecular formula	C_28_H_35_NO_6_	C_28_H_35_NO_6_
HRMS–ESI (*m/z*)		
found:	482.2547 [M + H]^+^	482.2562 [M + H]^+^
calcd:	482.2543 [C_28_H_35_NO_6_ + H]^+^	482.2543 [C_28_H_35_NO_6_ + H]^+^
UV (MeOH) λ_max_ (log ε)	202 (4.1), 214 (sh, 4.1), 234 (sh, 4.0), 313 (3.6), 350 (3.6)	200 (4.1), 214 (sh, 4.0), 231 (sh, 3.9), 312 (3.6), 349 (3.6)
[α]_D_^21^ (*c* 0.1, MeOH)	−75.2	−86.6
IR (KBr) ν_max_ (cm^−1^)	3414, 2930, 1658, 1620, 1423, 1385, 1182, 1087	3427, 2930, 1680, 1620, 1404, 1385, 1179, 1128

Streptoquinoline A (**1**): The molecular formula of **1** was elucidated as C_28_H_35_NO_6_ based on HRESIMS measurements. The ^13^C NMR spectrum (in DMSO-*d*_6_) showed 28 signals, which were classified into five methyl carbons, seven sp^3^ methylene carbons, two sp^3^ methine carbons, two sp^2^ methine carbons, two sp^3^ quaternary carbons, two oxygenated sp^3^ quaternary carbons, five sp^2^ quaternary carbons, and three carbonyl carbons from an analysis of the HMQC spectrum. The ^1^H NMR spectrum (in DMSO-*d*_6_) exhibited signals corresponding to five methyl protons, seven sp^3^ methylene protons, two sp^3^ methine protons, two sp^2^ methine protons, and two hydroxy protons. The connectivity of proton and carbon atoms was assigned from the HMQC spectrum (Table 2).

**Table 2:** ^1^H and ^13^C NMR chemical shifts in **1** and **2**.^a^

position	**1**		**2**	
	δ_C_	δ_H_ (*J* in Hz)	δ_C_	δ_H_ (*J* in Hz)

1	38.4 t	1.82 (1H, d, 11.7)	38.3 t	1.82 (1H, d, 9.3)
		0.97 (1H, m)		1.00 (1H, m)
2	18.0 t	1.58 (1H, m)	18.0 t	1.61 (1H, m)
		1.43 (1H, m)		1.44 (1H, m)
3	41.3 t	1.38 (1H, m)	41.3 t	1.40 (1H, m)
		1.15 (1H, m)		1.18 (1H, m)
4	32.8 s	–	32.8 s	–
5	55.2 d	1.04 (1H, d, 11.7)	55.1 d	1.06 (1H, m)
6	19.2 t	1.70 (1H, d, 13.7)	19.1 t	1.68 (1H, d, 13.2)
		1.38 (1H, m)		1.38 (1H, m)
7	39.5^a^ t	2.03 (1H, d, 11.7)	39.5^a^ t	2.03 (1H, d, 12.7)
		1.58 (1H, m)		1.60 (1H, m)
8	77.9 s	–	78.0 s	–
9	51.1 d	1.58 (1H, m)	51.1 d	1.59 (1H, m)
10	36.5 s	–	36.5 s	–
11	19.6 t	2.55^b^ (1H, overlap)	19.2 t	2.57 (1H, m)
		2.48^b^ (1H, overlap)		2.42 (1H, m)
12	33.1 q	0.89 (3H, s)	33.1 q	0.89 (3H, s)
13	21.4 q	0.83 (3H, s)	21.4 q	0.83 (3H, s)
14	14.5 q	0.91 (3H, s)	14.4 q	0.91 (3H, s)
15	20.0 q	1.15 (3H, s)	20.2 q	1.16 (3H, s)
1'	120.8 s	–	120.6 s	–
2'	131.6 s	–	131.6 s	–
3'	156.1 s	–	156.2 s	–
4'	73.4 s	–	73.5 s	–
4'-OH	–	6.22 (1H, s)		6.19 (1H, s)
5'	191.5 s	–	191.4 s	–
6'	143.8 s	–	143.9 s	–
7'	131.2 d	7.99 (1H, d, 7.8)	130.8 d	8.02 (1H, br s)
8'	124.2 d	7.95 (1H, d, 7.8)	124.1 d	7.94 (1H, br s)
9'	143.6 s	–	143.8 s	–
10'	165.8 s	–	165.8 s	–
10'-OOH	–	12.95 (1H, br s)		12.95 (1H, br s)
11'	51.2 t	4.02 (1H, d, 17.1)	51.0 t	3.83 (1H, d, 12.7)
		3.45 (1H, d, 17.1)		3.46 (1H, d, 12.7)
12'	206.9 s	–	207.0 s	–
13'	29.6 q	1.97 (3H, s)	29.8 q	1.99 (3H, s)

^a13^C NMR (100 MHz) and ^1^H NMR (400 MHz) spectra were taken on a JNM-AL-400 (JEOL) in DMSO-*d*_6_, and solvent peaks were used as internal standards at 2.49 ppm for ^1^H NMR and at 39.5 ppm for ^13^C NMR. ^b^Chemical shifts overlapping with solvent peaks were assigned based on 2D NMR correlations.

**Figure 2:**
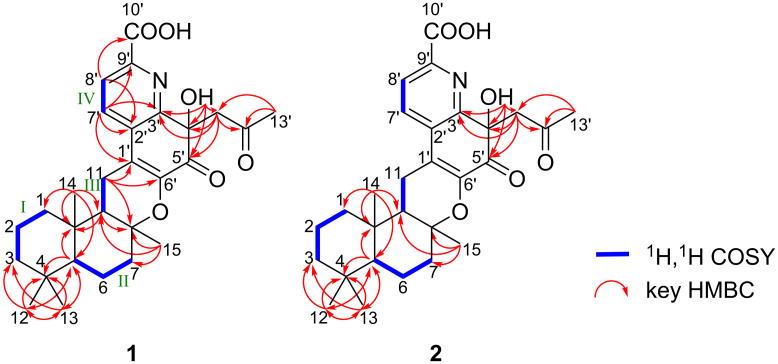
Structural elucidation of compounds **1** and **2** by 2D NMR experiments.

The planar structure of compound **1** was elucidated by a 2D NMR experiment, including ^1^H,^1^H COSY (bold line in Figure 2) and HMBC (arrow in Figure 2) correlations. Three partial structures I–III were initially identified based on ^1^H,^1^H COSY correlations: I (H_2_-1 (δ 1.82, 0.97)/H_2_-2 (δ 1.58, 1.43)/H_2_-3 (δ 1.38, 1.15)), II (H-5 (δ 1.04)/H_2_-6 (δ 1.70, 1.38)/H_2_-7 (δ2.03, 1.58)), and III (H-9 (δ 1.58)/H_2_-11 (δ 2.55, 2.48)). ^1^H,^13^C HMBC correlations were then observed from the methyl protons H_3_-12 (δ 0.89) and H_3_-13 (δ 0.83) to the sp^3^ methylene carbon C-3 (δ 41.3), the sp^3^ quaternary carbon C-4 (δ 32.8), and the sp^3^ methine carbon C-5 (δ 55.2). Correlations were observed from the methyl proton H_3_-14 (δ 0.91) to the sp^3^ methylene carbon C-1 (δ 38.4), C-5, the sp^3^ methine carbon C-9 (δ 51.1), and the sp^3^ quaternary carbon C-10 (δ 36.5) and from the methyl proton H_3_-15 (δ 1.15) to the sp^3^ methylene carbon C-7 (δ 39.5), the oxygenated sp^3^ quaternary carbon C-8 (δ 77.9), and C-9. These cross-peaks established connectivity among partial structures I–III, confirming a drimane-type sesquiterpene skeleton in compound **1**.

Additionally, ^1^H,^1^H COSY correlations confirmed the partial structure IV, consisting of two *ortho*-coupled aromatic protons, H-7' (δ 7.99) and H-8' (δ 7.95). HMBC correlations were observed from the sp^2^ methine proton H-7' (δ 7.99) to the sp^2^ quaternary carbon C-1' (δ 120.8), the sp^2^ quaternary carbon C-2' (δ 131.6), the nitrogenated sp^2^ quaternary carbon C-3' (δ 156.1), and the nitrogenated sp^2^ quaternary carbon C-9' (δ 143.6), from the sp^2^ methine proton H-8' (δ 7.95) to C-2', and the carboxylic carbon C-10' (δ 165.8); additionally, the hydroxy proton 4'-OH (δ 6.22) showed HMBC correlations to C-3', the oxygenated sp^3^ quaternary carbon C-4' (δ 73.4), and the ketone carbon C-5' (δ 191.5). These correlations clearly indicated the presence of a quinoline moiety. Furthermore, HMBC correlations were observed from the sp^3^ methylene proton H_2_-11' (δ 4.02, 3.45) to C-3', C-4', C-5', and the ketone carbon C-12' (δ 206.9) and from the methyl proton H_3_-13' (δ 1.97) to the sp^3^ methylene carbons C-11' (δ 51.2) and C-12', establishing the presence of an acetonyl group at C-4'.

HMBC correlations were observed from the sp^3^ methylene proton H_2_-11 to C-8, C-1', and the oxygenated sp^2^ quaternary carbon C-6' (δ 143.8). These correlations indicated that the quinoline unit was fused to a drimane-type sesquiterpene through two key links: a C–O bond between C-8 and C-6' and a C–C bond between C-11 and C-1'. Consequently, the planar structure of compound **1** was elucidated as shown in Figure 1, consistent with the molecular formula and degree of unsaturation.

**Figure 3:**
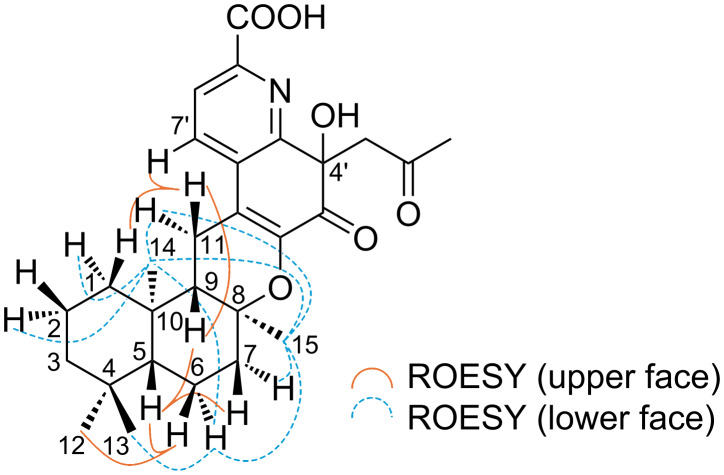
ROESY correlations of **1**.

The relative configuration of compound **1** was elucidated based on ROESY correlations, as shown in Figure 3. Clear ROESY cross-peaks were observed between H-5 and H-7 (δ 1.58)/H-9, as well as between H-6 (δ 1.70) and H_3_-12, indicating that these protons were located on the same face of the molecule. In contrast, the ROESY correlations between H-6 (δ 1.38) and the methyl protons H_3_-13, H_3_-14, and H_3_-15, together with cross-peaks between H_3_-14 and H_3_-15, suggested that these protons resided on the opposite face. These results support a relative configuration of 5R*, 8S*, 9S*, and 10R* for the drimane skeleton of compound **1**. However, it was not possible to elucidate the configuration at C-4' from available NMR data.

Streptoquinoline B (**2**): The molecular formula of compound **2** was elucidated as C_28_H_35_NO_6_ based on HRESIMS measurements. The ^13^C NMR and ^1^H NMR spectra (in DMSO-*d*_6_) of compound **2** showed chemical shift values that were almost identical to those of compound **1** (Table 2). Furthermore, 2D NMR experiments revealed similar ^1^H,^1^H COSY and HMBC correlations as those observed for **1** (Figure 2), confirming that compound **2** shared the same planar structure. The specific rotations of **1** and **2** were similar in both sign and magnitude, suggesting that they shared the same absolute configuration for most chiral centers. However, a notable difference was observed in the chemical shift of H-11' between the two compounds. This difference was consistent with an inversion at C-4', indicating that compounds **1** and **2** were epimers at this position.

Compounds **1** and **2** possessed a functional group at C-4' that appeared to be derived from acetone; therefore, they may be artifacts that formed via the nucleophilic addition of acetone to a ketone-bearing precursor during the extraction process. Despite various modifications to fermentation conditions, it was not possible to detect or isolate the presumed natural precursor. We are currently undertaking the total synthesis of this putative natural precursor to further confirm its structure and biological properties.

To date, only two structurally related meroterpenoids to **1** and **2** have been reported: thallusin [7] and saccharoquinoline [8], both of which share a drimane-type sesquiterpene skeleton. Thallusin was isolated from the marine bacterium *Zobellia uliginosa*, while saccharoquinoline was obtained from *Saccharomonospora* sp., another marine-derived actinomycete. Notably, compounds **1** and **2** represent the first examples of drimane–quinoline-type meroterpenoids produced by a terrestrial *Streptomyces* strain, and their structural relationship to thallusin-type sesquiterpenes suggests a biosynthetic connection; however, the quinoline moieties in **1** and **2** were more highly substituted.

The specific optical rotations of **1** ([α]_D_ −75.2) and **2** ([α]_D_ −86.6) were negative and similar in magnitude to that of synthetic (−)-thallusin sodium salt ([α]_D_ −82.4) [9], suggesting that their drimane cores shared the same absolute configuration (5*R*,8*S*,9*S*,10*R*). In contrast, saccharoquinoline has been reported to exhibit a positive specific optical rotation ([α]_D_ +55) [8], indicating that its drimane core is the enantiomer of those found in **1**, **2**, and (−)-thallusin.

The antibacterial activities of **1** and **2** were evaluated by the microdilution method in accordance with the CLSI guideline M07-A11 [10]. The minimum inhibitory concentrations (MICs) of **1** and **2** against nine test microorganisms are summarized in Table 3. Compounds **1** and **2** both exhibited antibacterial activity against Gram-positive bacteria, including VRE and MRSA, with MICs ranging from 6.25 to 12.5 µg/mL. These results highlight their potential as lead compounds for the development of new antibacterial agents targeting drug-resistant Gram-positive pathogens. In contrast, no inhibitory activity was observed against Gram-negative bacteria (*Escherichia coli* and *Pseudomonas aeruginosa*) or mycobacteria (*Mycobacterium bovis* BCG and *M. avium*) even at 200 µg/mL, indicating that compounds **1** and **2** possess a relatively narrow, but specific antibacterial spectrum.

**Table 3:** MICs of **1** and **2**.

Test microorganism	MICs (µg/mL)		

	**1**	**2**	control

*Enterococcus faecalis*	6.25	6.25	2.0^a^
Vancomycin-resistant *E. faecalis*	6.25	6.25	>256^a^
Multidrug-resistant *E. faecium*	12.5	12.5	>256^a^
*Staphylococcus aureus*	12.5	12.5	0.50^a^
Methicillin-resistant *S. aureus*	12.5	6.25	1.0^a^
*Escherichia coli*	>200	>200	0.0078^b^
*Pseudomonas aeruginosa*	>200	>200	0.13^b^
*Mycobacterium bovis* BCG	>200	>200	0.0020^c^
*M. avium*	>200	>200	0.25^c^

^a^Vancomycin. ^b^Ciprofloxacin. ^c^Rifampicin.

Previously reported related meroterpenoids, such as thallusin and saccharoquinoline, exhibited no antibacterial biological activities – thallusin acts as an inducer of morphogenesis in some marine algae [7], whereas saccharoquinoline exhibits cytotoxicity towards the HCT-116 cancer cell line through G1 arrest [8]. Therefore, the discovery of antibacterial activity by compounds **1** and **2** expands the known biological potential of drimane–quinoline-type meroterpenoids beyond their previously recognized roles.

## Conclusion

Streptoquinolines A (**1**) and B (**2**) are novel meroterpenoids featuring a drimane-type sesquiterpene fused with a highly substituted quinoline unit. These compounds were isolated from terrestrial *Streptomyces* sp. TMPU-A0679 and both exhibited selective antibacterial activity against Gram-positive pathogens, including VRE and MRSA. Structural and stereochemical analyses established that **1** and **2** shared the same planar structure and drimane core configuration (5*R*,8*S*,9*S*,10*R*), but differed in their stereochemistries at C-4', indicating that they are epimers. Based on the presence of an acetone-derived substituent at C-4', these compounds may be artifacts of a ketone-bearing precursor formed during extraction. The discovery of streptoquinolines expands the structural and biological diversities of drimane–quinoline-type meroterpenoids and provides novel insights into their potential as scaffolds for the development of antibacterial agents targeting multidrug-resistant Gram-positive bacteria.

## Experimental

### Materials

Ciprofloxacin, rifampicin, and vancomycin were purchased from FUJIFILM Wako Pure Chemical Industries (Osaka, Japan). Yeast extract, malt extract, and peptone were obtained from Becton, Dickinson and Company (Franklin Lakes, NJ, USA). Ehrlich meat extract was purchased from Kyokuto Pharmaceutical Co. (Tokyo, Japan). All other chemicals used in this study were of special grade.

### General experimental procedures

HRESIMS spectra were recorded using a mass spectrometer (Xevo G2-XS QTof, Waters Co., Milford, MA, USA). NMR spectra were acquired on a spectrometer (JNM-AL-400; JEOL, Tokyo, Japan).

### Microorganisms

The actinomycete strain TMPU-A0679 was identified as *Streptomyces* sp. from its 16S rDNA sequence in a BLAST search by TechnoSuruga Laboratory (Shizuoka, Japan). The following strains were used in antibacterial activity assays: *E. faecalis* ATCC 29212, vancomycin-resistant *E. faecalis* ATCC 51575, multidrug-resistant *E. faecium* ATCC 700221, *S. aureus* NBRC 13276, methicillin-resistant *S. aureus* ATCC 700698, *E. coli* NBRC 3972, *P. aeruginosa* NBRC 13275, *M. bovis* BCG Pasteur, and *M. avium* JCM 15430.

### Fermentation

In a manner similar to our previous report [5], an agar plate culture of strain TMPU-A0679 grown on ISP medium No. 2 (yeast extract 0.4%, malt extract 1.0%, glucose 0.4%, and agar 1.5%; adjusted to pH 7.3) was used to inoculate a 500 mL Erlenmeyer flask containing 100 mL of the seed medium (potato starch 2.4%, yeast extract 0.5%, glucose 0.1%, peptone 0.3%, Ehrlich meat extract 0.3%, and CaCO_3_ 0.4%; adjusted to pH 7.0). The flask was incubated on a rotary shaker at 180 rpm at 27 °C for 4 days. One milliliter of the seed culture was transferred into sixty 500 mL Erlenmeyer flasks containing 100 mL of ISP2 medium No. 2. Fermentation was conducted on a rotary shaker (Iwashiya Bio Science, Tokyo, Japan) at 180 rpm at 27 °C for 7 days.

### Isolation of compounds **1** and **2**

The culture broth (6.0 L) was centrifuged to separate the mycelia and supernatant. Collected mycelia were treated with acetone (1.0 L) for 60 minutes. After suction filtration, the acetone solution was concentrated under reduced pressure to remove the solvent. The resulting aqueous solution (400 mL) was extracted twice with EtOAc (400 mL × 2, pH 3). The combined EtOAc layer was dried over Na_2_SO_4_ and concentrated under reduced pressure to yield crude material (922 mg). This material was then dissolved in a small amount of MeOH, applied to an ODS (Fuji Silysia Chemical Ltd., Aichi, Japan) column (i.d. 20 × 170 mm), and eluted with MeOH aq. solvent system in a stepwise manner (20 mL × 6 fractions for each solvent, 0, 20, 40, 60, 80, and 100% MeOH, acetone). The 80% MeOH aq. eluate was concentrated to give crude materials containing **1** and **2** (23.5 mg). These materials were finally purified by preparative HPLC: column, Develosil C30-UG (Nomura Chemical Co., Ltd., Aichi, Japan), i.d. 10 × 250 mm; mobile phase, 55% MeCN aq. isocratic; detection, UV at 210 nm; flow rate, 3.0 mL /min. Under these conditions, compounds **1** and **2** eluted with retention times of 37 and 34 min, respectively. Each eluate was concentrated and dried in vacuo to yield pure samples of **1** (7.50 mg) and **2** (8.05 mg), respectively, as a yellow oil.

### Assessment of MICs using the broth microdilution method

Antibacterial activity was evaluated using the broth microdilution method in accordance with CLSI guideline M07-A11 [10]. Bacterial strains were cultured at 37 °C overnight in Mueller–Hinton broth (Becton Dickinson, San Jose, CA, USA). Overnight cultures were diluted with the same broth and adjusted to an optical density of 0.0548 at 550 nm (approximately 1.0 × 10^8^ CFU/mL). The suspensions were then further diluted 3,000-fold with fresh broth. Aliquots of 95 µL of the diluted suspension were dispensed into the wells of a 96-well microplate, followed by the addition of 5 µL of test samples dissolved in methanol. The microplate was incubated at 37 °C for 24 hours. Bacterial growth was assessed by measuring turbidity at 550 nm using a spectrophotometer. The MIC was defined as the lowest concentration of the test compound that inhibited ≥90% of bacterial growth relative to the control (no compound).

## Supporting Information

File 1 MS and NMR spectra of streptoquinolines A (**1**) and B (**2**).

## Data Availability

All data that supports the findings of this study is available in the published article and/or the supporting information of this article.
