# Supplementary material for: Streptoquinolines A and B, new antibacterial meroterpenoids produced by Streptomyces sp. TMPU-A0679
Source: Beilstein J Org Chem. 2026 Jan 27;22:185–91. doi: 10.3762/bjoc.22.12 (PMC12862604; doi:10.3762/bjoc.22.12)
Supplement: File 1 — MS and NMR spectra of streptoquinolines A (1) and B (2). [file Beilstein_J_Org_Chem-22-185-s001.pdf]

BEILSTEIN JOURNAL OF ORGANIC CHEMISTRY

Supporting Information

for

Streptoquinolines A and B, new antibacterial meroterpenoids produced by *Streptomyces* sp. TMPU-A0679

Akiho Yagi, Hitomi Tomura, Ami Konno and Ryuji Uchida

Beilstein J. Org. Chem. **2026,** *22,* 185–191. doi:10.3762/bjoc.22.12

MS and NMR spectra of streptoquinolines A (1) and B (2)

License and Terms: This is a supporting information file under the terms of the Creative Commons Attribution License (https://creativecommons.org/licenses/by/4.0). Please note that the reuse, redistribution and reproduction in particular requires that the author(s) and source are credited and that individual graphics may be subject to special legal provisions.
The license is subject to the *Beilstein Journal of Organic Chemistry* terms and conditions: (https://www.beilstein-journals.org/bjoc/terms)

Table of contents

Figure S1: ESI-MS spectrum of **1** S2

Figure S2: ^{1}H NMR spectrum of **1** (400 MHz, DMSO-d_6) S2

Figure S3: ^{13}C NMR spectrum of **1** (100 MHz, CD_3OD) S3

Figure S4: HMQC spectrum of **1** (400 MHz, CD_3OD) S3

Figure S5: COSY spectrum of **1** (400 MHz, CD_3OD) S4

Figure S6: HMBC spectrum of **1** (400 MHz, CD_3OD) S4

Figure S7: ROESY spectrum of **1** (400 MHz, CD_3OD) S5

Figure S8: ESI-MS spectrum of **2** S5

Figure S9: ^{1}H NMR spectrum of **2** (400 MHz, DMSO-d_6) S6

Figure S10: ^{13}C NMR spectrum of **2** (100 MHz, CD_3OD) S6

Figure S11: HMQC spectrum of **2** (400 MHz, CD_3OD) S7

Figure S12: COSY spectrum of **2** (400 MHz, CD_3OD) S7

Figure S13: HMBC spectrum of **2** (400 MHz, CD_3OD) S8

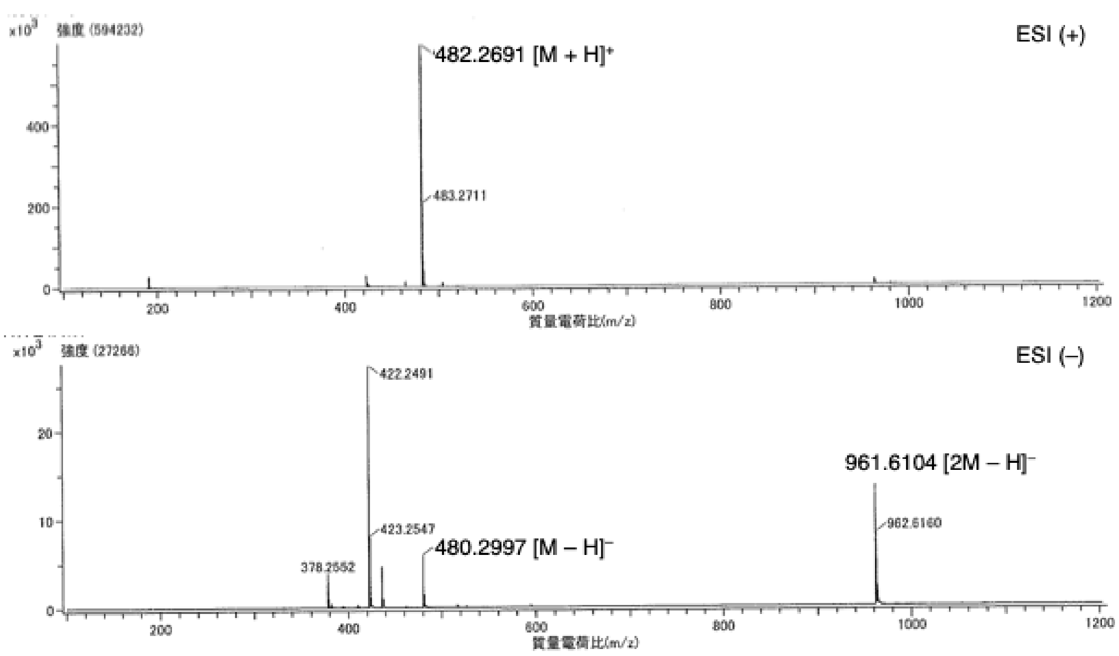

Figure S1: ESI-MS spectrum of **1**.

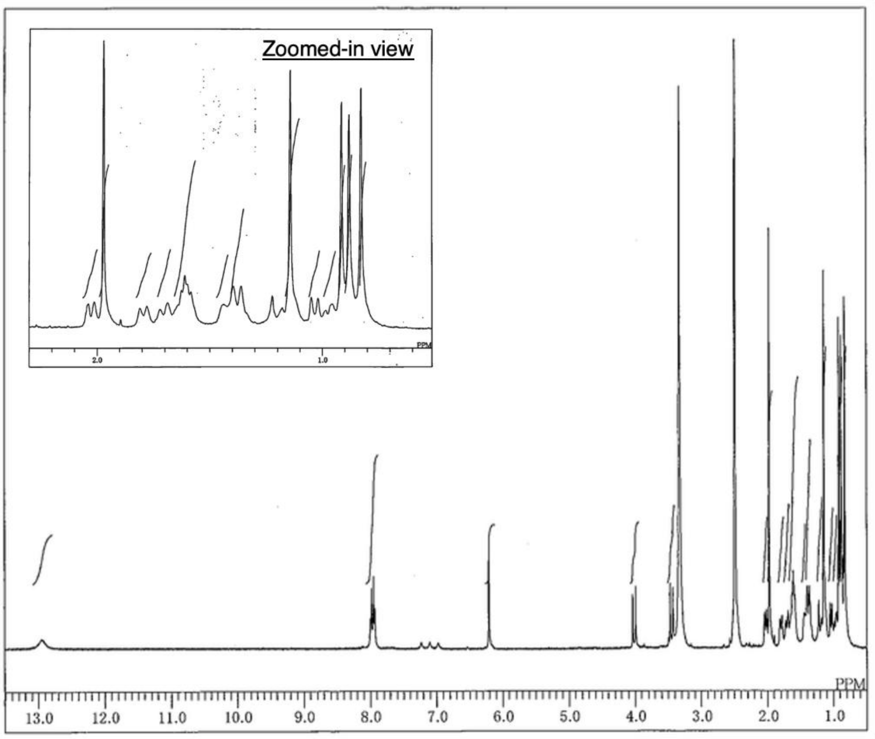

Figure S2: ^{1}H NMR spectrum of **1** (400 MHz, DMSO-d_6).

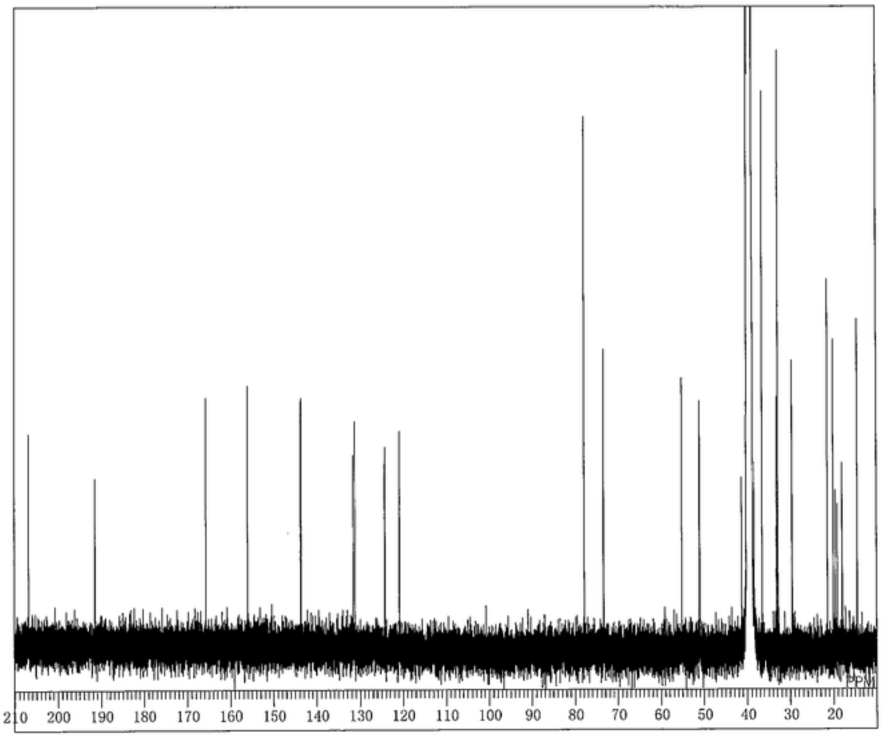

Figure S3: ^{13}C NMR spectrum of **1** (100 MHz, DMSO-d_6).

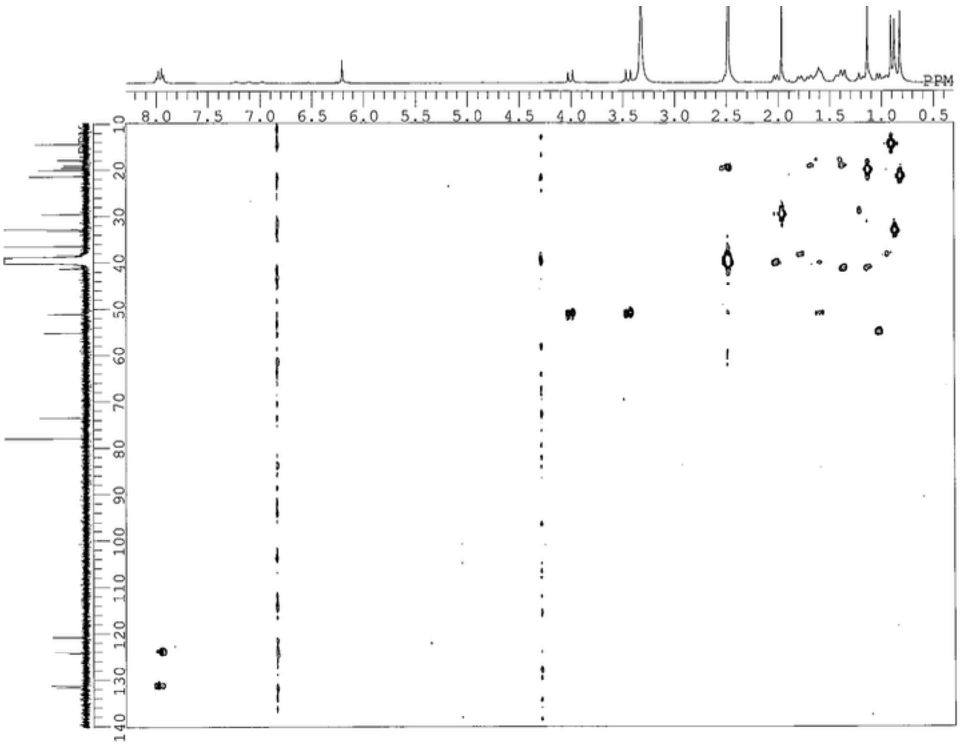

Figure S4: HMQC spectrum of **1** (400 MHz, DMSO-d_6).

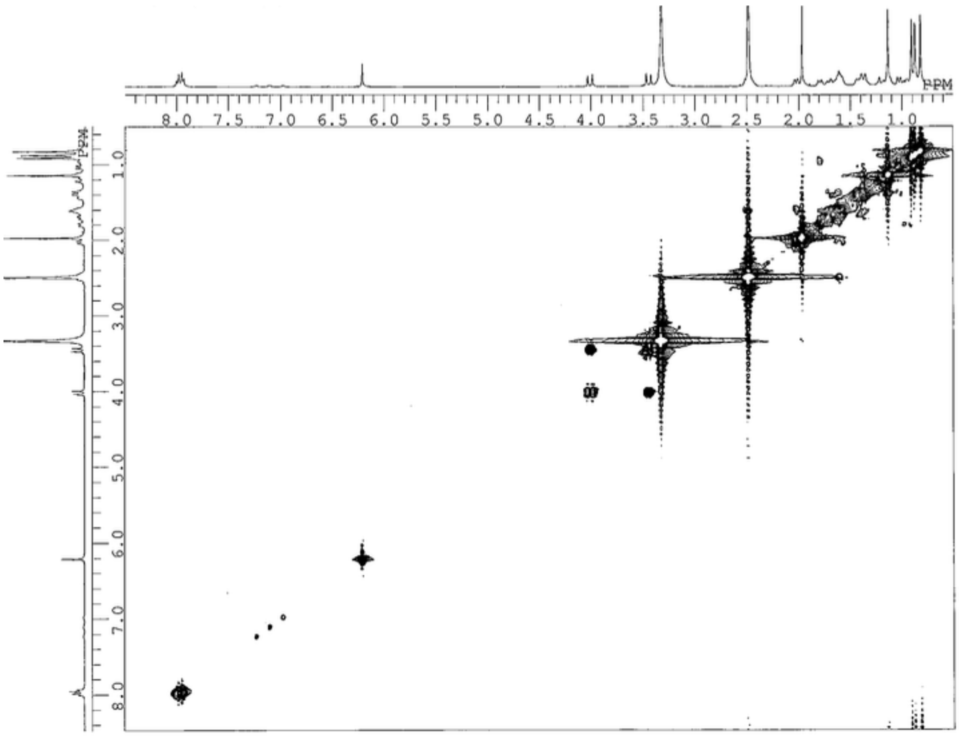

Figure S5: COSY spectrum of **1** (400 MHz, DMSO-d_6).

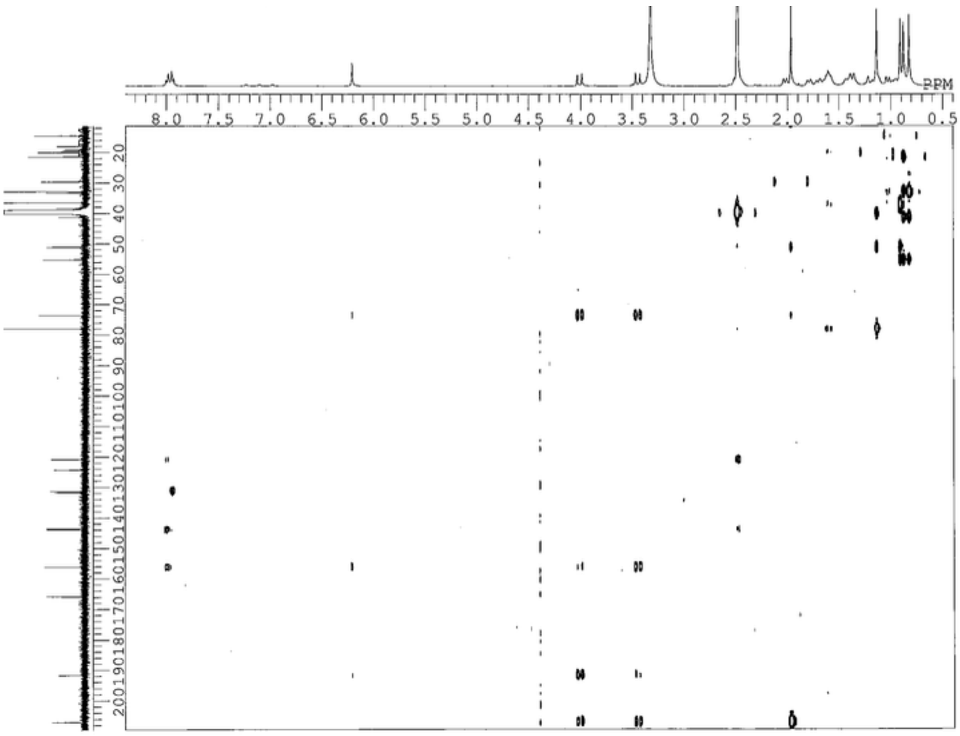

Figure S6: HMBC spectrum of **1** (400 MHz, DMSO-d_6).

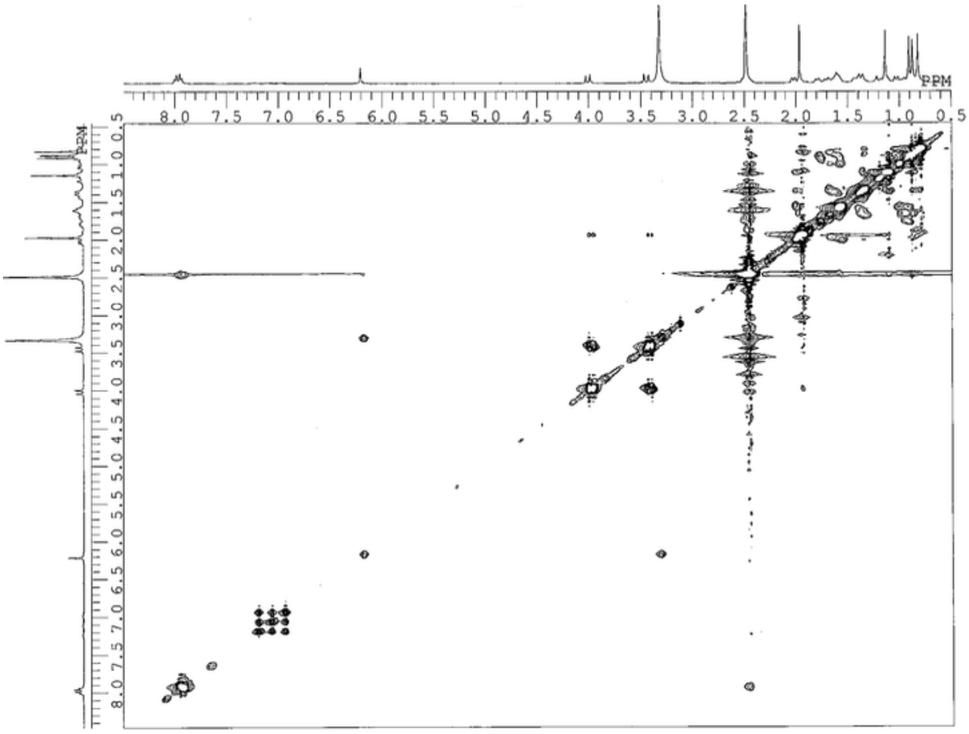

Figure S7: ROESY spectrum of **1** (400 MHz, DMSO-d_6).

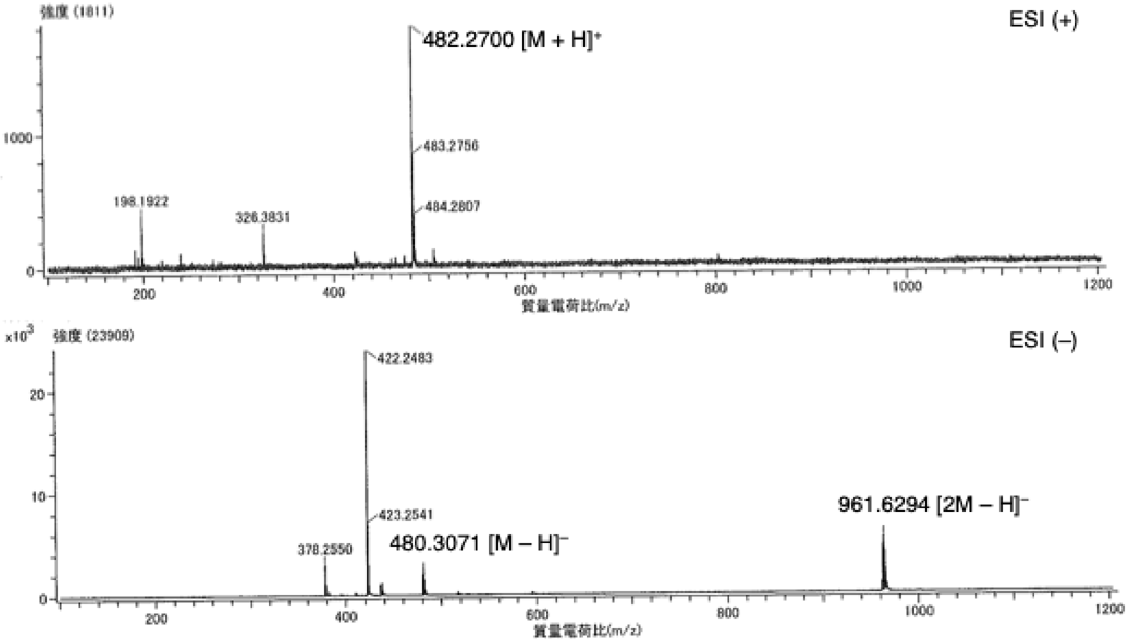

Figure S8: ESI-MS spectrum of **2**.

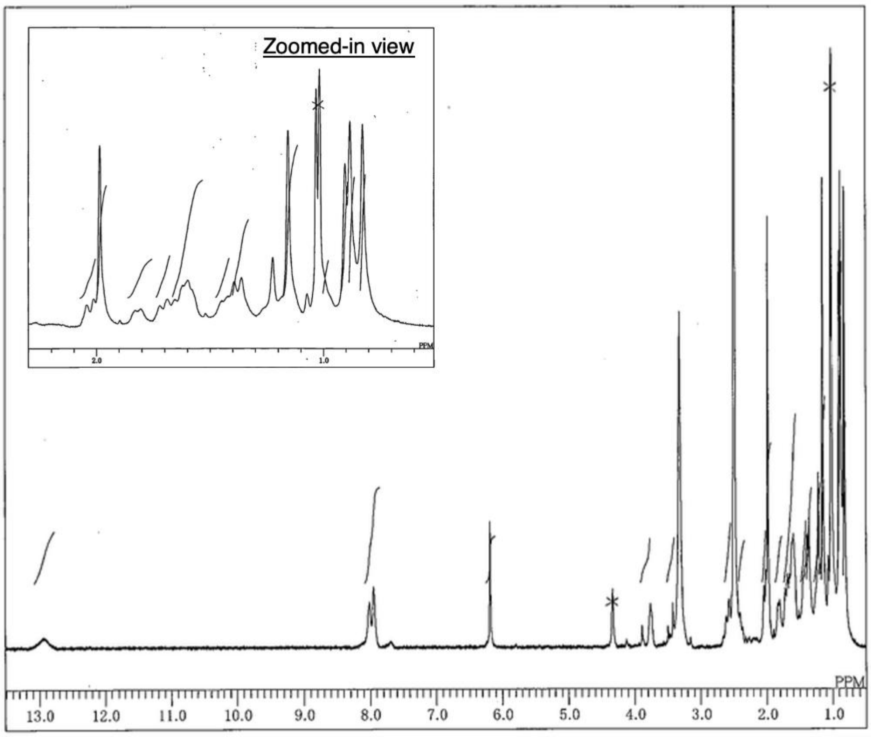

Figure S9: ^{1}H NMR spectrum of **2** (400 MHz, DMSO-d_6).

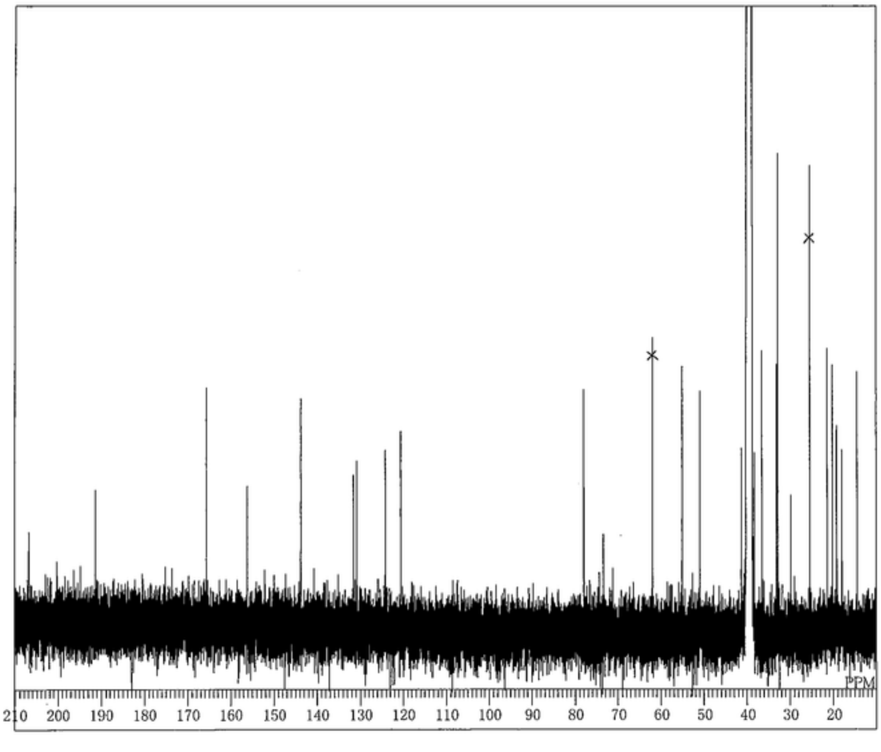

Figure S10: ^{13}C NMR spectrum of **2** (100 MHz, DMSO-d_6).

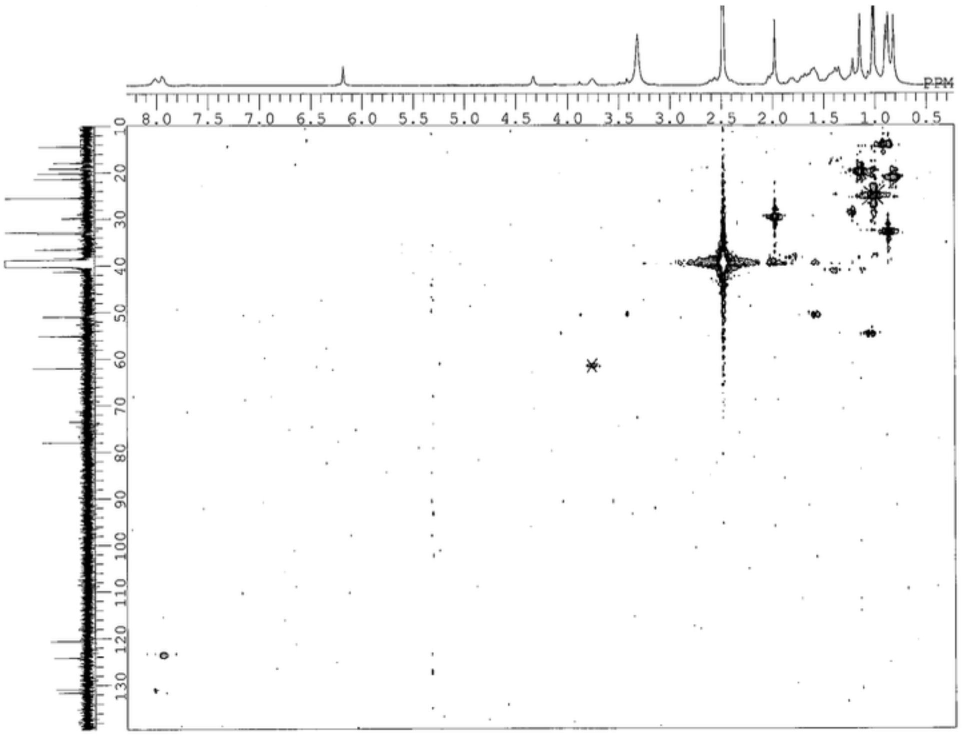

Figure S11: HMQC spectrum of **2** (400 MHz, DMSO-d_6).

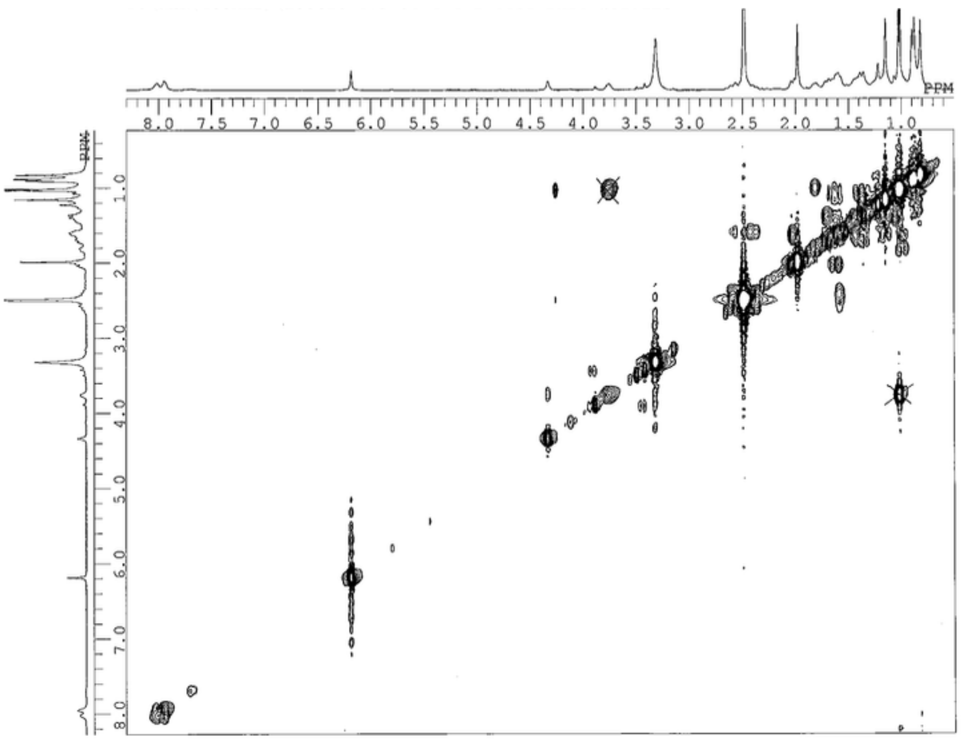

Figure S12: COSY spectrum of **2** (400 MHz, DMSO-d_6).

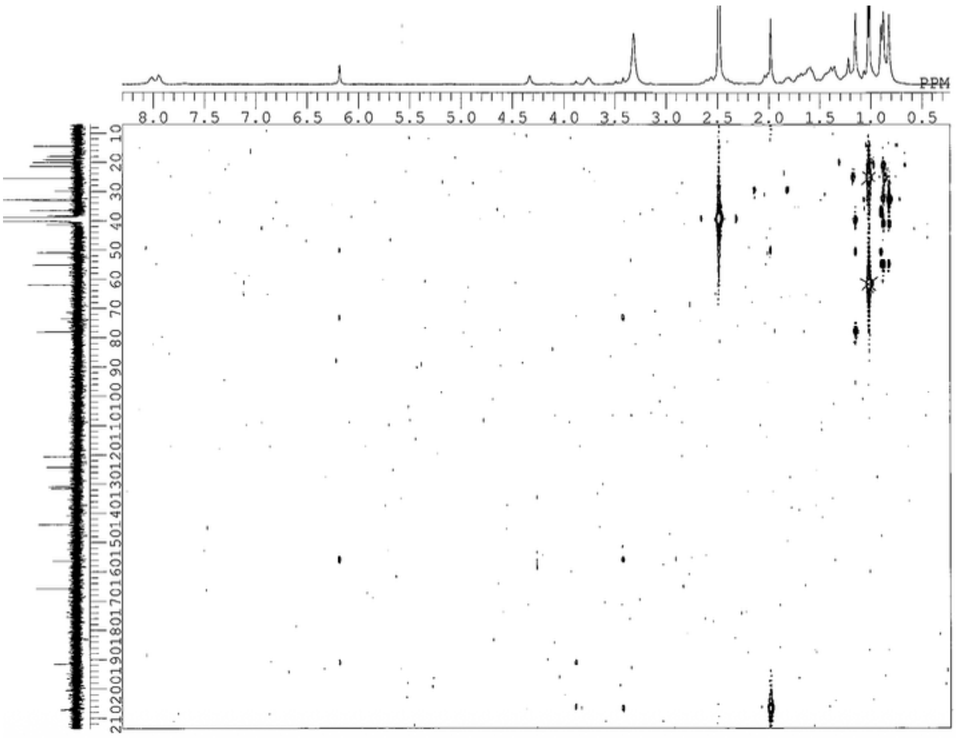

Figure S13: HMBC spectrum of **2** (400 MHz, DMSO-d_6).
